# Assessment of renal function in mice with unilateral ureteral obstruction using ^99m^Tc-MAG3 dynamic scintigraphy

**DOI:** 10.1186/1471-2369-13-168

**Published:** 2012-12-10

**Authors:** Mohammed N Tantawy, Rosie Jiang, Feng Wang, Keiko Takahashi, Todd E Peterson, Dana Zemel, Chuan-Ming Hao, Hiroki Fujita, Raymond C Harris, Christopher C Quarles, Takamune Takahashi

**Affiliations:** 1 Radiology and Vanderbilt University Institute of Imaging Science, Nashville, TN, USA; 2 Vanderbilt O'Brien Mouse Kidney Physiology and Disease Center, Vanderbilt University Medical Center, Nashville, TN, USA; 3 Division of Endocrinology, Metabolism and Geriatric Medicine, Akita University Graduate School of Medicine, Akita, Japan; 4 Division of Nephrology and Hypertension, Vanderbilt University School of Medicine, S-3223, MCN, Nashville, TN, 37232, USA

**Keywords:** Nuclear imaging, Renal scintigraphy, ^99m^Tc-MAG3, Mouse model, Unilateral ureteral obstruction, Renal function

## Abstract

**Background:**

Renal scintigraphy using ^99m^Tc-mercaptoacetyltriglycine (^99m^Tc-MAG3) is widely used for the assessment of renal function in humans. However, the application of this method to animal models of renal disease is currently limited, especially in rodents. Here, we have applied ^99m^Tc-MAG3 renal scintigraphy to a mouse model of unilateral ureteral obstruction (UUO) and evaluated its utility in studying obstructive renal disease.

**Methods:**

UUO mice were generated by complete ligation of the left ureter. Sham-operated mice were used as a control. Renal function was investigated on days 0, 1, 3, and 6 post-surgery using dynamic planar imaging of ^99m^Tc-MAG3 activity following retro-orbital injection. Time-activity curves (TACs) were produced for individual kidneys and renal function was assessed by 1) the slope of initial ^99m^Tc-MAG3 uptake (SIU), which is related to renal perfusion; 2) peak activity; and 3) the time-to-peak (TTP). The parameters of tubular excretion were not evaluated in this study as ^99m^Tc-MAG3 is not excreted from UUO kidneys.

**Results:**

Compared to sham-operated mice, SIU was remarkably (>60%) reduced in UUO kidneys at day 1 post surgery and the TACs plateaued, indicating that ^99m^Tc-MAG3 is not excreted in these kidneys. The plateau activity in UUO kidneys was relatively low (~40% of sham kidney’s peak activity) as early as day1 post surgery, demonstrating that uptake of ^99m^Tc-MAG3 is rapidly reduced in UUO kidneys. The time to plateau in UUO kidneys exceeded 200 sec, suggesting that ^99m^Tc-MAG3 is slowly up-taken in these kidneys. These changes advanced as the disease progressed. SIU, peak activity and TTPs were minimally changed in contra-lateral kidneys during the study period.

**Conclusions:**

Our data demonstrate that renal uptake of ^99m^Tc-MAG3 is remarkably and rapidly reduced in UUO kidneys, while the changes are minimal in contra-lateral kidneys. The parametric analysis of TACs suggested that renal perfusion as well as tubular uptake is reduced in UUO kidneys. This imaging technique should allow non-invasive assessments of UUO renal injury and enable a more rapid interrogation of novel therapeutic agents and protocols.

## Background

There is considerable interest and potential value in making available imaging methods that permit non-invasive assessments of renal function in animals. Renal scintigraphy or renograms using ^99m^Tc-mercaptoacetyltriglycine (^99m^Tc-MAG3) is widely used for the assessment of renal function in humans, especially for the evaluation of postoperative complications in renal transplant recipients [1]. This radiotracer is extracted from the kidneys with high efficiency through secretion from proximal tubules [2,3]. The time-activity curves (TACs) of ^99m^Tc-MAG3 renograms, which are produced by dynamic scan, enable the real-time and quantitative evaluation of multiple features of renal function, while the renography created by static scan provides spatial information about ^99m^Tc-MAG3 kinetics and kidney morphology. The first phase of the TACs, which rises as ^99m^Tc-MAG3 flows into renal tissue, can be used as an indicator of renal perfusion and tubular uptake, while the descending phase provides an assessment of tubular excretion [4,5] (Additional file 1). Thus, the measurement of intrarenal ^99m^Tc-MAG3 kinetics provides a minimally invasive, simple, and quantitative test of renal function, which does not require blood or urine sampling. Given these features, ^99m^Tc-MAG3 renography could serve as a useful tool for the repetitive and longitudinal assessment of renal function in pre-clinical animal models of renal disease. However, the application of this method to animal models of renal disease is currently limited, especially in rodents. In mice, only a few studies have employed it to assess ischemia and reperfusion renal injury [5,6]. Also, quantitative analysis and longitudinal investigation of ^99m^Tc-MAG3 renograms have not been carried out in animals with unilateral ureteral obstruction (UUO).

Obstructive nephropathy is a major cause of renal impairment in infants and children [7]. Over the past few decades, the cellular and molecular events involved in obstructive renal injury have been elucidated by surgical ligation of a ureter (known as UUO) in postnatal and adult animals [8,9]. These include tubular and myofibroblast proliferation, interstitial inflammation, tubular atrophy/apoptosis, interstitial fibrosis, and renin-angiotensin and TGFβ activation. Further, the UUO model recapitulates all of the key features of renal fibrosis, a hallmark of progressive renal disease; therefore, the model is widely used for the research of renal fibrogenic response [9,10]. However, disorders of renal function during the course of the UUO model are incompletely understood, as the measurements of renal function in a single kidney are methodologically limited. Thus, the purpose of the present study was to investigate the use of dynamic ^99m^Tc-MAG3 imaging for the assessment of renal function in a mouse UUO model. Our data demonstrate that renal uptake of ^99m^Tc-MAG3 is remarkably and rapidly reduced in UUO kidneys as early as day 1 post-surgery, while it is minimally changed in contralateral kidneys. Further, the parametric analysis of TACs suggested that renal perfusion as well as tubular uptake is reduced in UUO kidneys. This imaging technique should allow non-invasive assessments of UUO renal injury and enable a more rapid interrogation of novel therapeutic agents and protocols.

## Methods

### Animals

Male C57BL/6N mice, aged 6 weeks, were purchased from Harlan Laboratories (Indianapolis, IN). Animals were fed a standard diet and allowed free access to water. All animal procedures were conducted in accordance with institutional guidelines and approved by the Vanderbilt University Institution of Animal Care and Use Committee.

### Unilateral ureteral obstruction

Mice were anesthetized with 2% isoflurane and the left kidney was exposed through the site of the left flank incision. The ureter was obstructed completely near the renal pelvis using a 4–0 silk tie suture at two points. Sham-operated mice underwent the same surgical procedure except for the ureter ligation. The mice were imaged at 0 (3 hours), 1, 3, and 6 days after surgery.

### ^99m^Tc-MAG3 Renal scintigraphy

Mice were anesthetized with 1.5%/98.5% isoflurane/oxygen mixture and two mice at a time were placed directly on top of a parallel-hole collimator positioned over one of the four camera heads of a NanoSPECT/CT (Bioscan, Washington, DC). The NanoSPECT was set up in single-camera planar-dynamic mode. A 40 min dynamic acquisition was initiated simultaneously with retro-orbital injection of ~37 MBq/0.2 mL of ^99m^Tc-MAG3 (obtained from VUMC Radiopharmacy, Vanderbilt University). The 40 min dynamic planar acquisition was divided into 240 frames yielding a temporal resolution of 10 sec. Two-dimensional images were collected in a 256 x 256 matrix with a pixel size of 0.29 mm x 0.29 mm. For static imaging, three dimensional images were collected in a 176 x 176 x 112 matrix with a pixel size of 0.2 mm x 0.2 mm x 0.2 mm through a 40-min scan (a temporal resolution of 40 min), producing high resolution imaging data. In a comparison study (retro-orbital vs. intravenous injections), ^99m^Tc-MAG3 was injected into mice via jugular catheter. Jugular vein catheterization was carried out as described [11].

### Image analysis

The planar images were imported into the medical image viewer software Amide [12]. Two-dimensional ellipsoidal shaped regions of interest (ROIs) were drawn around each kidney and a background region outside the mouse. The radioactivity within the ROIs was extracted for each frame and normalized to the injected dose for each animal, dividing image voxel values by the actual injected dose as measured in a CRC-15W dose calibrator (Capintec, Ramsey, NJ), to establish the TACs over the entire duration of the scan. The data were expressed as normalized image value (NIV). The slope of initial linear uptake (from 10s to 40s)(SIU) in units of 1/s, peak activity in units of NIV, and the time to peak or plateau (TTP or TTPt) in units of seconds were computed from the renogram of each kidney [5,13]. [Note: Since the TACs of UUO kidneys plateau and do not peak, we measured the plateau activity and the time-to-plateau for these kidneys.]

### Magnetic resonance imaging (MRI)

Structural changes in UUO kidneys were assessed by MRI studies on a Varian 7T horizontal bore imaging system (Lexington, MA). Anesthesia was induced and maintained with a 1.5%/98.5% isoflurane/oxygen mixture. A constant body temperature of 37°C was maintained using heated air flow. Animals were placed in a prone position and imaged using a birdcage 25-mm inner diameter radiofrequency (RF) coil. All imaging protocols were based on a field-of-view of 25.6 x 25.6 mm^2^. A multi-slice gradient echo imaging sequence [repetition time (TR) =25ms; echo time (TE) = 2.5ms; 128 x 128 matrix; 2-mm slice thickness] was used to acquire 3-plane multi-slice images for proper positioning and slice selection. A coherent gradient echo sequence was used for T1-weighted imaging (TR = 45 ms; TE = 2.25 ms; flip angle = 35°; number of excitations (NEX) = 81). T2 contrast was achieved with the use of a fast spin-echo (FSE) sequence (TR = 2000ms; effective TE = 48 ms; RARE-factor 8, 256 x 256 matrix; 0.5-mm slice thickness).

### Histological assessment

Kidneys were removed from euthanized mice*,* fixed overnight in 10% buffered formalin, and paraffin tissue sections were stained with Masson trichrome using standard procedures.

### Statistical analysis

One-way ANOVA followed by a Dunnett post test was carried out to compare the mean values in different days and groups. A two-tailed paired t-test was carried out for comparisons between obstructed and unobstructed (contralateral) kidneys. Data were expressed as means ± SEM. The derived *p*-values were considered statistically significant when less than 0.05.

## Results

In the present study, we assessed renal function in UUO mice using ^99m^Tc-MAG3 dynamic renal scintigraphy. Renal uptake of ^99m^Tc-MAG3 was assessed by parametric analysis of TACs. Since ^99m^Tc-MAG3 is not excreted from UUO kidneys, parametric analysis of ^99m^Tc-MAG3 excretion was not performed in this study.

### Renal structure and histopathology in UUO mice

**Figure 1:**
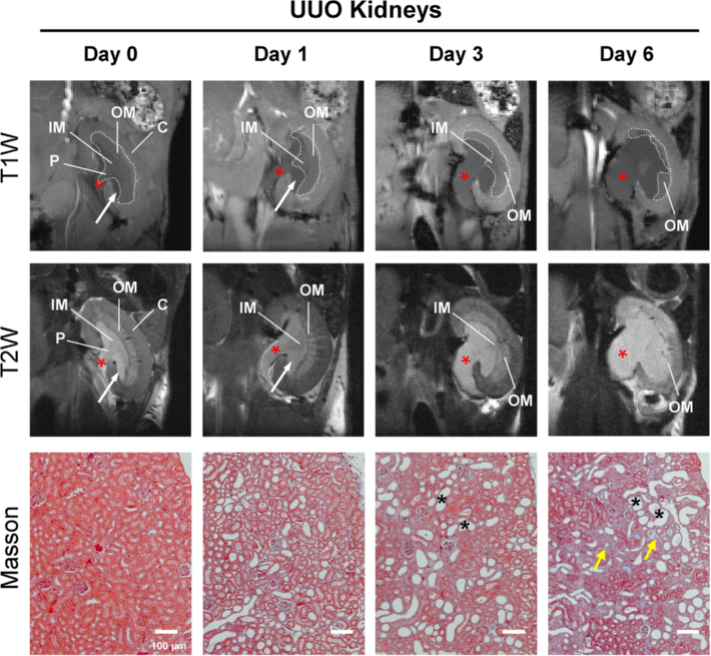
**Renal structure and histopathology in UUO mice.** (**A**) Representative T1- and T2-weighted coronal MRI images of UUO kidney at day 0, 1, 3 and 6 post obstruction. Renal pelvic space (asterisks) is progressively expanded in the obstructed kidney, and renal medulla (outlined with dotted lines), especially inner medulla and papilla, is remarkably decreased as the disease progresses. Arrows indicate urine. C, cortex; OM, outer medulla (inner stripe); IM, inner medulla; P, papilla. (**B**) Representative histological sections of mouse kidneys subjected to unilateral ureteral obstruction. Tubulointerstitial region increases markedly over 6 days in UUO kidneys, as indicated by destructive morphology of renal tubules (asterisks) and interstitial fibrosis (arrows). Bar=100 μm. Masson trichrome stain

First, we confirmed generation and severity of UUO model by MRI and histological investigation. Figure 1 shows representative T1- and T2-weighted MR images of a UUO mouse at 0, 1, 3 and 6 days post-surgery. The renal pelvic space was gradually expanded in obstructed kidneys as early as 1 day post ureteral ligation and the components of renal medulla, especially inner medulla and papilla, were remarkably decreased as the disease progressed. Reduction of renal cortex was also observed at day 3 and day 6 post obstruction. Histological investigation displayed typical patterns of UUO renal disease, including tubular dilatation, flattening and atrophy, and expansion of interstitial area with extracellular matrix accumulation and infiltration of inflammatory cells (Figure 1, bottom panels). Tubulointerstitial region progressed markedly over 6 days after ureteral obstruction. Obvious structural changes were not observed in contralateral kidneys and sham-operated mouse kidneys (data not shown).

### Parametric analysis of TACs and comparison of intravenous and retro-orbital ^99m^Tc-MAG3 injections

**Figure 2:**
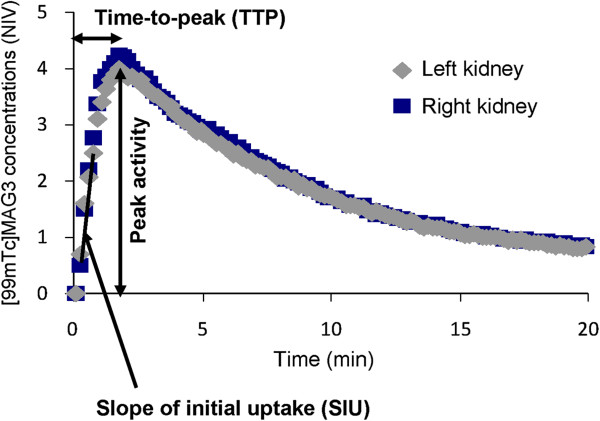
**Representative time**-**activity curves (TACs) of **^**99m**^**Tc-MAG3 dynamic renal scintigraphy in normal mice. **^99m^Tc-MAG3 was retro-orbitally injected to mice and the TACs were established as described in “Materials and Methods”. The slope of initial uptake (SIU) which rises linearly, peak activity, and the time-to-peak (TTP) were determined and used as parameters of renal ^99m^Tc-MAG3 uptake

**Figure 3:**
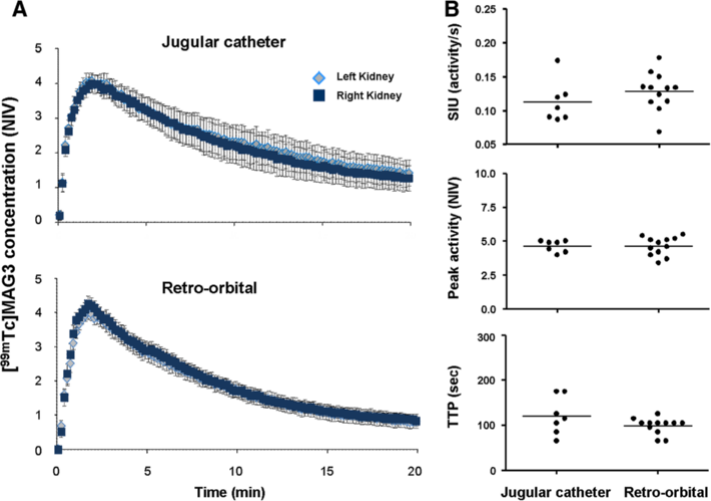
**Comparison of TACs established by intravenous and retro**-**orbital **^**99m**^**Tc-MAG3 injections**. (**A**) Mean TACs established by intravenous (n=7) and retro-orbital (n=12) ^99m^Tc-MAG3 injections. For intravenous injection, ^99m^Tc-MAG3 was injected via jugular vein catheter. Values are means ± SE. (**B**) Comparison of the parameters (SIU, peak activity, and TTP) extracted from the TACs generated by intravenous and retro-orbital ^99m^Tc-MAG3 injections. No significant difference was found between the two procedures

Renal function was assessed in UUO kidneys based on the TACs and their parametric analysis. Three parameters were measured for individual kidneys: 1) the slope of initial linear uptake (SIU, 1/s), which is related to renal perfusion; 2) the peak activity (NIV); and 3) the time-to-peak or plateau (TTP or TTPt, sec) (Figure 2). Radiotracers are usually administrated via the tail vein in rodents. However, repeated injections are difficult as the procedure frequently causes soft tissue extravasations and tail scars, preventing subsequent injections. Repeatable and proper intravenous injections are fundamental for the longitudinal and quantitative analysis of ^99m^Tc-MAG3 renal scintigraphy. Therefore, we first compared retro-orbital and intravenous injections, given the facts that the former is much easier to accomplish at multiple time points and that retro-orbital injection was recently shown to be an effective route of radiotracer administration during PET studies in mice [14]. As shown in Figure 3A, the TACs of ^99m^Tc-MAG3 renal scintigraphy obtained from retro-orbital injections were comparable to those from intravenous injections via jugular vein catheter. There was no significant difference in the SIU, peak activity, and TTP parameters between these two procedures (Figure 3B). In addition, a comparative study using UUO day 1 mice also showed no difference between these two administration routes (data not shown). These results demonstrate that retro-orbital injection can be used for ^99m^Tc-MAG3 renal scintigraphy in mice. Therefore, in this study we assessed renal function in UUO mice using retro-orbital injections of ^99m^Tc-MAG3.

### Assessment of renal function in UUO mice using ^99m^Tc-MAG3 dynamic scintigraphy

**Figure 4:**
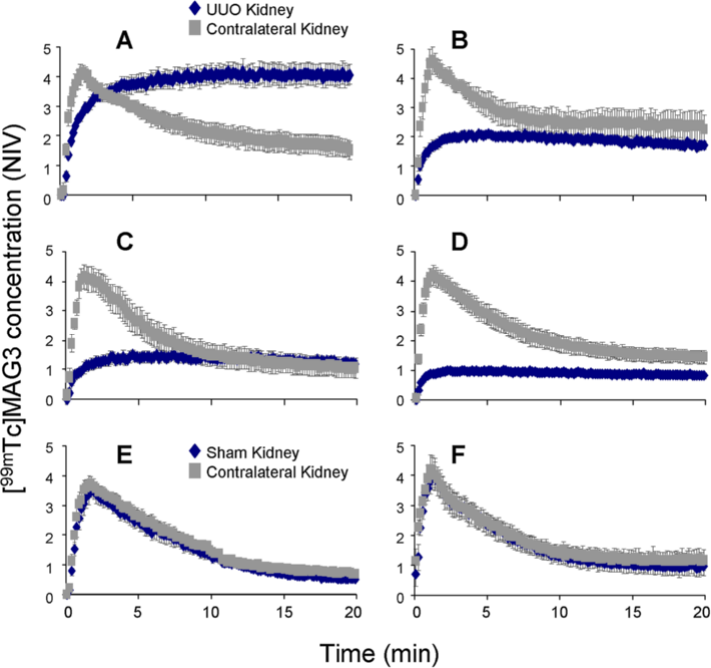
**TACs of **^**99m**^**Tc-MAG3 dynamic renal scintigraphy in UUO mice.** (**A**) UUO day 0, (**B**) UUO day 1, (**C**) UUO day 3, (**D**) UUO day 6, (**E**) Sham operation day 0, (**F**) Sham operation day 6. Values are means ± SE. The numbers of mice are described in Table 1

**Figure 5:**
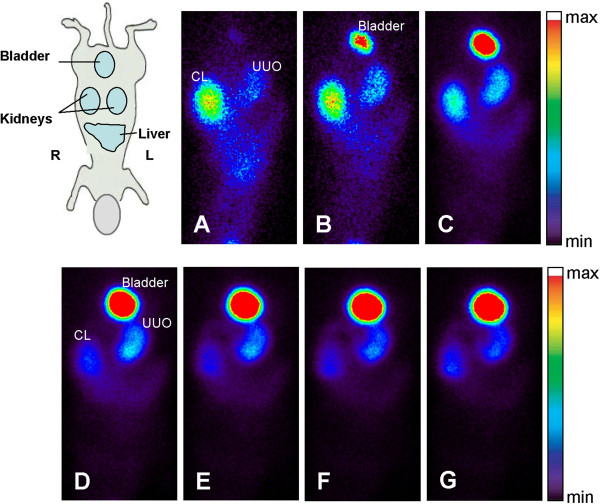
**Representative time**-**interval images of **^**99m**^**Tc-MAG3 dynamic renal scintigraphy in UUO mice at 6 days post obstruction.** Uptake of the tracer is remarkably reduced in UUO kidney. **A**) 0–1 min, **B**) 1–2 min, **C**) 2–6 min, **D**) 6–15 min, **E**) 15–25 min, **F**) 25–35 min, and **G**) 35–40 min. R, right; L, left; CL, contralateral kidney

**Figure 6:**
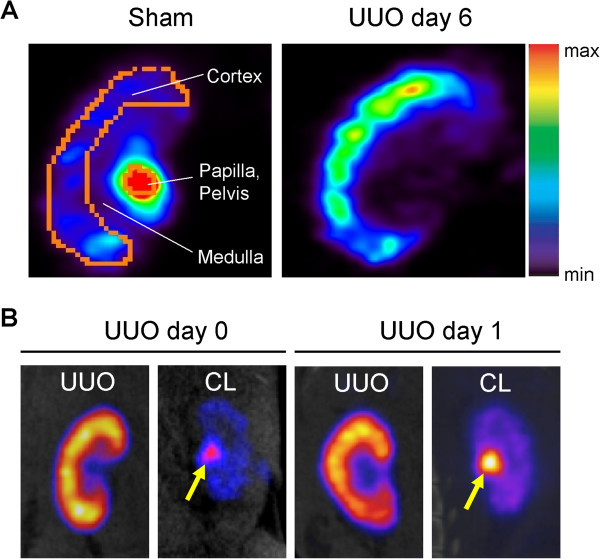
**Static **^**99m**^**Tc-MAG3 imaging of UUO mice kidneys. A**) Static SPECT images (a 40-min acquisition) were obtained from UUO day 6 and sham-operated mice kidneys after retro-orbital injection of ^99m^Tc-MAG3. The excretion of ^99m^Tc-MAG3 to renal pelvis is not observed in UUO day 6 kidney, while ^99m^Tc-MAG3 gets into renal pelvis in sham-operated kidney during a 40 min scan. **B**) Similar findings were observed in UUO day 0 and day 1 kidneys, while ^99m^Tc-MAG3 was excreted to renal pelvis (arrows) in contralateral kidneys (CL). Representative images are shown

**Figure 7:**
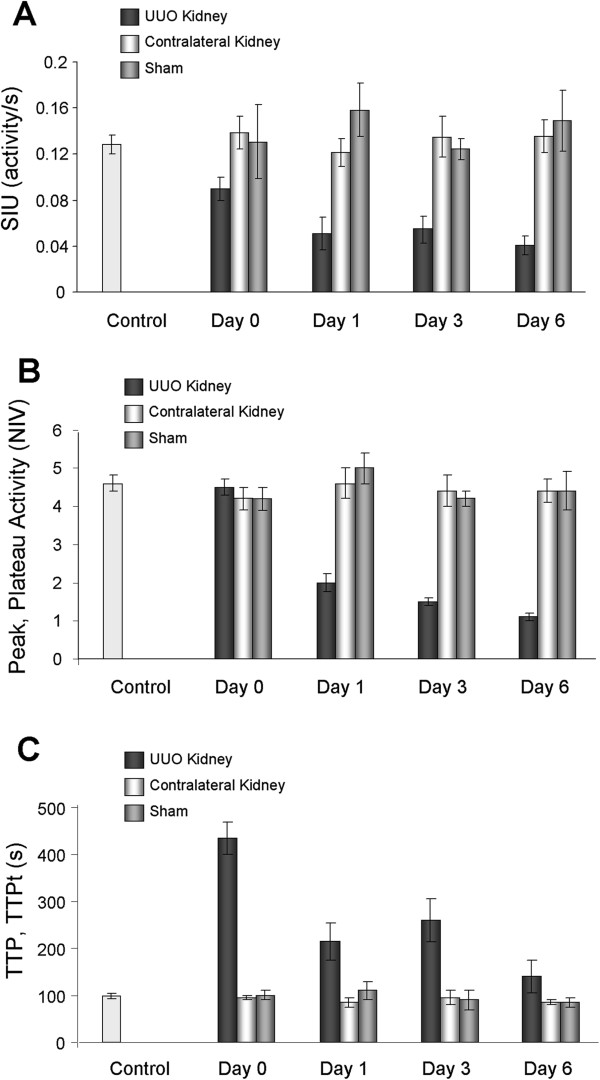
**Parametric analysis of **^**99m**^**Tc-MAG3 dynamic renal scintigraphy obtained from UUO, sham**-**operated**, **and non-surgery (control) mice.** Values are means ± SE. The numbers of mice are described in Table 1. Note: The time-to-plateau (TTPt) was measured in UUO kidneys

**Figure 8:**
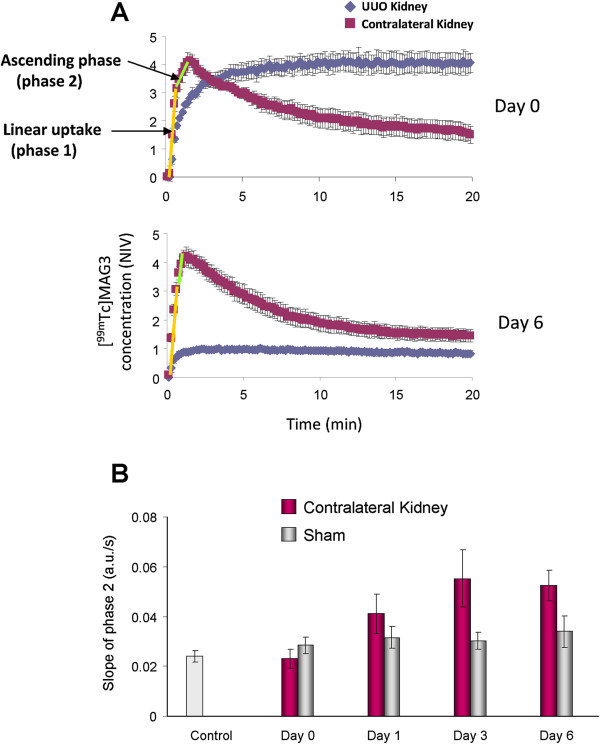
**Slope of ascending phase is increased in contralateral kidneys after UUO surgery. A**) Graphical depiction of ascending phase window selection in contralateral kidneys at 0 and 6 days post ureteral obstruction. **B**) The slope of ascending phase (from 50s to peak) was measured for the contralateral kidneys in UUO and sham-operated mice. Values are means ± SE. The numbers of mice are described in Table 1

Renal scintigraphy was carried out in UUO and sham-operated mice at 0 (3 hours), 1, 3, and 6 days after surgery. Non-surgery mice were also imaged as a control. Figure 4 A-D shows TACs of UUO kidneys at each time point. As shown in Figure 4B, renal uptake of ^99m^Tc-MAG3 was remarkably reduced in UUO kidneys as early as day 1 post obstruction, as indicated by low total activity throughout the TACs, and it was further decreased as the disease progressed (Figure 4C and 4D). The TACs in UUO kidneys plateaued over time, indicating that ^99m^Tc-MAG3 is not excreted in these kidneys. Profound reduction of ^99m^Tc-MAG3 uptake was also demonstrated in UUO kidney by the time interval images of renal scintigraphy (Figure 5). Further, static renal scintigraphy demonstrated that excretion of ^99m^Tc-MAG3 to renal pelvis is highly limited in UUO kidneys, even at day 0 (3 hrs) post surgery (Figure 6); the finding indicates that TACs of UUO kidneys mostly demonstrate the deposition of ^99m^Tc-MAG3 in renal parenchyma. The contralateral kidneys in UUO mice (Figure 4 A-D) and kidneys in sham-operated mice (Figure 4E and 4F) showed similar TACs. Table 1 and Figure 7 show the parametric analysis of ^99m^Tc-MAG3 renal scintigraphy based on TACs obtained from UUO and sham operation mice at day 0, 1, 3, and 6 post surgery. The results of statistical analysis are shown in Table 2. As compared to sham-operated kidneys, SIU was remarkably (> 60%) decreased in UUO kidneys as early as day 1 post obstruction, while contralateral kidneys showed SIU values comparable to those in sham-operated kidneys (Figure 7A). It is noteworthy that the slope of the ascending phase (50s to peak, denoted Phase 2), which follows the initial linear uptake of ^99m^Tc-MAG3 (10s to 40s, denoted Phase 1), was progressively increased in contralateral kidneys, though it was not statistically significant against day 0 or sham operation kidneys (Figure 8). The plateau activity of TACs was also remarkably (~65%) reduced in UUO kidneys as early as day 1 post obstruction and it declined further as the disease progressed, while the peak activity in contralateral kidneys was comparable to those in sham-operated kidneys during the study period (Figure 7B). Further, the time-to-plateau (TTPt) in UUO kidneys largely exceeded the time-to-peak (TTP) values in sham-operated or contralateral kidneys (Figure 7C), suggesting that ^99m^Tc-MAG3 is slowly up-taken in UUO kidneys. The TTP was unaltered in contralateral kidneys as compared with sham operation kidneys, yet contralateral kidneys at UUO day 6 showed significantly shorter TTP than at UUO day 0, 1, and 3 (Figure 7C, Table 2). There was no significant difference in the peak:10min ratio, an indicator of tubular excretion rate [5,6], between contralateral kidneys and sham-operated kidneys, though contralateral kidneys showed lower peak:10min ratios at day 0 and day 1, perhaps due to intravascular fluid shifts during surgical procedure (data not shown). No difference was observed in SIU, peak activity, TTP, and peak:10min ratio between sham-operated and non-surgery mice kidneys and between sham-operated and contralateral kidneys in sham mice.

**Table 1:** Parameters extracted from the TACs of ^99m^ Tc-MAG3 dynamic renal scintigraphy

**Kidney**	**n**	Peak or plateau * activity (NIV)	**SIU (activity/s)**	**TTP**, TTPt* (s)
Normal (non-surgery)	12	4.63 ± 0.20	0.129 ± 0.008	98 ± 5
UUO day 0	6	4.48 ± 0.19	0.090 ± 0.010	437 ± 35
UUO day 1	5	1.98 ± 0.20	0.051 ± 0.014	213 ± 38
UUO day 3	6	1.45 ± 0.10	0.055 ± 0.011	258 ± 46
UUO day 6	8	1.06 ± 0.09	0.041 ± 0.008	138 ± 33
Contralateral day 0	6	4.22 ± 0.26	0.139 ± 0.015	93 ± 5
Contralateral day 1	5	4.56 ± 0.36	0.122 ± 0.012	91 ± 9
Contralateral day 3	6	4.43 ± 0.35	0.135 ± 0.018	95 ± 14
Contralateral day 6	8	4.43 ± 0.28	0.136 ± 0.014	83 ± 5
Sham day 0	7	4.19 ± 0.33	0.131 ± 0.032	101 ± 10
Sham day 1	8	4.95 ± 0.40	0.158 ± 0.023	108 ±18
Sham day 3	5	4.16 ± 0.18	0.125 ± 0.009	87 ± 17
Sham day 6	4	4.35 ± 0.49	0.149 ± 0.027	85 ± 7

Values are means ± SE. * Plateau activity and the time-to-plateau (TTPt) were measured in UUO kidneys.

**Table 2:** **Summary of statistical tests between obstructed**, **contralateral**, **and sham**-**operated kidneys on all days post UUO**/**sham surgery**

**Test**	**P <0.05**		
	**Peak activity**	**SIU**	**TTP**
UUO vs. Sham	days 1, 3, 6	days 1, 3, 6	N/A*
Contralateral vs. Sham	none	none	none
UUO vs. Contralateral	days 1, 3, 6	days 0, 1, 3, 6	N/A*
UUO vs. UUO	day 0 vs. days 1, 3 & 6; day 1 vs. day 6	day 0 vs. day 6	day 0 vs. days 1, 3 & 6
Contralateral vs. Contralateral	none	none	day 6 vs. days 0, 1, & 3

* As TACs of UUO kidney did not peak, statistical test was not applied to this section.

## Discussion

In the present study, we have applied ^99m^Tc-MAG3 renal scintigraphy to a mouse model of obstructive nephropathy and non-invasively evaluated renal function over 6 days post obstruction. Our results demonstrate that renal uptake of ^99m^Tc-MAG3 is rapidly reduced by ureteral ligation, while it is minimally changed in the contralateral kidney. This work provides the first non-invasive evaluation of longitudinal changes in renal function in UUO animals. Further, our data demonstrate that retro-orbital injection can be used for ^99m^Tc-MAG3 renal scintigraphy in mice.

A substantial reduction of SIU was observed in UUO kidney. Several studies have demonstrated acute reduction in renal blood flow (RBF) in UUO animals. Vaughan et al. measured RBF in a dog UUO model using a flow probe placed in renal artery and showed that RBF is transiently increased in UUO kidney at 0–1.5 hrs post obstruction, followed by a progressive decline [15,16]. In their study, RBF was decreased by ~50% in UUO kidney within 18 hrs post obstruction, while changes in RBF were not observed in contralateral kidney. Similar results have been demonstrated by other studies measuring renal arterial blood flow in rat or dog UUO models [17-19]. Thus, the changes of SIU are consistent with these previous results of RBF measurements on renal artery in experimental UUO kidneys, suggesting that the decreases in SIU may largely result from reduced renal perfusion. It is of note that the plateau activity is also substantially reduced in UUO mouse kidney at day 1 post obstruction, whereas a reduction of vessel density is not observed at this time point; the finding suggests that the capacity of ^99m^Tc-MAG3 uptake is also reduced in UUO day 1 kidney, perhaps due to tubular cell injury. This may also be the reason why the time-to-plateau is shortened in UUO kidney at day 1 post obstruction. Thus, our results suggest that reduced renal perfusion and tubular injury causes remarkable reduction of ^99m^Tc-MAG3 uptake in UUO kidney and that these parameters may be used for assessing the severity of obstructive renal disease.

In contrast to the obstructed kidney, the changes in SIU, peak activity, and TTP were not observed in contralateral kidney, even though the obstructed kidney was functionally dead. Given the fact that unilateral nephrectomy increases renal arterial blood flow by ~50% in two days [20,21], it seems that the systemic factors produced by the UUO kidney and/or the presence of renal blood flow in the obstructed kidney attenuate adaptive responses in the contralateral kidney. It is of note that the slope of the ascending phase after linear uptake of ^99m^Tc-MAG3 was increased in contralateral kidneys. Although the mechanism of this is currently unknown, the finding suggests that early tubular excretion of ^99m^Tc-MAG3 may be decreased in contralateral kidneys.

In dog and rat UUO models, it has been suggested that increased renal endothelin-1 production [18], enhanced renin-angiotensin system (RAS) [22], and insufficient nitric oxide synthesis [15,16,23] cause vasoconstriction in obstructed kidney, and these alterations can increase renal vascular resistance, especially pre-glomerular resistance, resulting in reduction of renal arterial blood flow. Pharmacological agents to antagonize endothelin or RAS system, L-arginine to promote nitric oxide production, and a calcium channel blocker (verapamil) which suppresses vasoconstriction were shown to be effective in increasing renal arterial blood flow in UUO kidney [16,18,19,22-24]. It would be of interest to determine the roles of these pathways in reduction of ^99m^Tc-MAG3 uptake in a mouse UUO model using this imaging technique and genetically manipulated mice or pharmacological treatments. Also, further investigation would be required to determine the changes of renal perfusion in a mouse UUO model using a nuclear or MR imaging technique (e.g. dynamic susceptibility contrast MRI) and evaluate the relation between renal perfusion and the uptake of ^99m^Tc-MAG3. Further, it is of note that tracer deposition within renal pelvis is highly limited in UUO mouse kidney, even at day 0 (3 hrs) post surgery. Similar findings were also reported in pig UUO kidney [25], These findings suggest that ureteral obstruction immediately reduces tubular function and the radiotracer does not get into the renal pelvis. Further investigation would be required to determine the mechanism of this. Last, compared to the obstructed kidney, much less is known about the adaptive responses and mechanisms in contralateral kidney. This issue should also be addressed in future study.

## Conclusions

This work assessed renal function in UUO mice by dynamic SPECT imaging with retro-orbital injection of ^99m^Tc-MAG3. Our data demonstrate that renal uptake of ^99m^Tc-MAG3 is remarkably and rapidly reduced in UUO kidneys, while the changes are minimal in contra-lateral kidneys. The parametric analysis of TACs suggested that renal perfusion as well as tubular uptake is reduced in UUO kidneys. Given the fact that similar changes are observed in ^99m^Tc-MAG3 renogram in human patients with renal obstruction [26,27], combination of this imaging technique with mouse UUO models should provide an effective assay for evaluating treatment protocols of this disease or screening new pharmacological agents as well as for elucidating pathophysiological mechanisms of obstructive kidney disease.

## Abbreviations

MAG3: Mercaptoacetyltriglycine; UUO: Unilateral ureteral obstruction; TACs: Time-activity curves; RBF: Renal blood flow; TTP: The time-to-peak; TTPt: The time-to-plateau; SPECT: Single photon emission-computed tomography; MRI: Magnetic resonance imaging.

## Competing interests

The authors have no competing interests to declare.

## Authors’ contributions

M.N.T., C.C.Q., and T.T. provided conception and design of research; M.N.T. and F.W. performed SPECT and MR imaging; R.J., K.T., and C.M.H. performed UUO and sham surgery; R.J., K.T., and H.F. performed histological analysis; M.N.T., D.Z., and T.E.P. analyzed data; M.N.T., R.C.H., C.C.Q., and T.T. interpreted results of experiments; M.N.T. and T.T. prepared figures and drafted the manuscript; T.E.P., R.C.H., C.C.Q., and T.T. edited and finalized the manuscript. All authors read and approved the final manuscript.

## Pre-publication history

The pre-publication history for this paper can be accessed here:

http://www.biomedcentral.com/1471-2369/13/168/prepub

## Supplementary Material

Additional file 1 **Tantawy MN et al_Supplemental Figure S1: A supplemental figure demonstrating the phases of **^**99m**^**Tc-MAG3 TACs and their determinants.** Click here for file
