# Supplementary material for: Assessment of renal function in mice with unilateral ureteral obstruction using 99mTc-MAG3 dynamic scintigraphy
Source: BMC Nephrol. 2012 Dec 10;13:168. doi: 10.1186/1471-2369-13-168 (PMC3542003; doi:10.1186/1471-2369-13-168)
Supplement: Additional file 1 — Tantawy MN et al_Supplemental Figure S1: A supplemental figure demonstrating the phases of 99mTc-MAG3 TACs and their determinants. [file 1471-2369-13-168-S1.pdf]

Tantawy MN et al. Additional File 1

Phase	Determinants
1. Linear uptake;	renal perfusion<br>tubular uptake<br>glomerular filtration*
2. Ascending phase;	renal perfusion<br>tubular uptake<br>glomerular filtration*<br>early tubular excretion
3. Descending phase;	tubular excretion

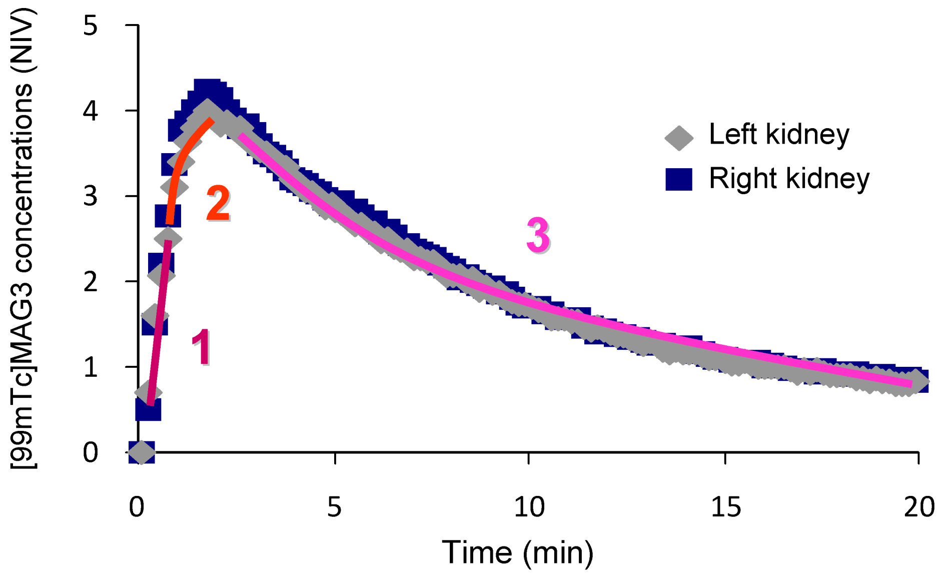

Figure S1 Phases of ^{99m}Tc-MAG3 TACs and the factors involved in each phase. * Glomerular filtration of ^{99m}Tc-MAG3 is minimum because of its high protein binding [Eshima D, Taylor A Jr. Semin Nucl Med. 1992 22(2):61-73].
